# GPX4 in the Tumor Microenvironment: Not Just Inhibiting Ferroptosis, but Immuno-Metabolic Regulation

**DOI:** 10.3390/biom16071006

**Published:** 2026-07-10

**Authors:** Xinzhe Li, Manxuan Zhang, Zenan Xu, Reziyamu Wufuer, Wenfang Li

**Affiliations:** 1 School of Pharmacy & Institute of Materia Medica, Xinjiang University, Urumqi 830017, China; lixinzhe@stu.xju.edu.cn (X.L.); zhangmanxuanxj@gmail.com (M.Z.); xuzenan@stu.xju.edu.cn (Z.X.); 2 College of Life Science and Technology, Xinjiang University, Urumqi 830046, China

**Keywords:** GPX4, ferroptosis, tumor microenvironment, immuno-metabolic checkpoint, lipid peroxidation threshold, cancer immunotherapy, spatial heterogeneity, therapeutic resistance

## Abstract

Glutathione peroxidase 4 (GPX4) is canonically viewed as the primary suppressor of ferroptosis, yet its role in the tumor microenvironment (TME) extends far beyond antioxidant catalysis to encompass immuno-metabolic regulation. In this review, we synthesize recent advances in enzymology, immunology, and cancer metabolism to propose a “lipid peroxidation threshold” framework, wherein GPX4 sets cell-type-specific thresholds that determine susceptibility to ferroptosis across tumor cells, CD8^+^ T cells, dendritic cells (DCs), and myeloid populations. We discuss how these thresholds are dynamically adjusted by post-translational modifications, nutrient competition and intercellular feedback loops, resulting in significant spatial heterogeneity between the tumor core and the tumor invasive front. There is a current selectivity paradox in GPX4 inhibitors, as well as resistance through nuclear factor erythroid 2-related factor 2 (Nrf2) and ferroptosis suppressor protein 1 (FSP1) that restricts the efficacy of GPX4 inhibitors as monotherapy. We focus on rational combination approaches: GPX4 modulation with immune checkpoint blockade (ICB), chemotherapy, and targeting myeloid-derived suppressor cells (MDSCs); and the pressing need for predictive biomarkers and single-cell spatial profiling. We conclude that successful clinical translation requires moving beyond indiscriminate GPX4 inhibition toward precision “threshold engineering” that selectively lowers tumor lipid peroxidation thresholds while sparing immune cells.

## 1. Introduction

GPX4 is a unique mammalian enzyme that reduces membrane lipid peroxides. Mammals possess seven other glutathione peroxidases (GPXs), but only GPX4 reduces complex lipid hydroperoxides following their insertion into cell membranes and the lipoproteins of the bloodstream [1,2,3]. GPX4 is indispensable for mammalian development, and germline deletion is embryonically lethal due to uncontrolled membrane lipid peroxidation, which cannot be compensated for by other antioxidant enzymes [4].

Conversely, the mevalonate pathway supports GPX4 maturation by isopentenylation of selenocysteine (Sec) tRNA. This forms a vital link between cholesterol synthesis and ferroptosis resistance, but the precise mechanism for this is still unclear [5]. More recently, palmitoyltransferase ZDHHC8 (zinc-finger DHHC-type) has been discovered to tether GPX4 closer to cell membranes of malignant cells, increasing local enzyme activity without changing overall enzyme levels [6]. Limited nutrients, low oxygen, and increased oxidative stress—the very same factors that fuel malignant adaptations—also modulate GPX4. Tissue-specific GPX4 deletion drives divergent pathologies from neurodegeneration to immune dysfunction, reflecting cell-type-specific physiological roles [7]. A Warburg-metabolizing tumor cell in a lactate-rich, hypoxic niche possesses a different lipid peroxidation challenge from an effector T cell that invades the same environment [8]. Yet, most studies explore GPX4 in monocultures lacking these important contextual cues [9,10,11].

Cystine, the rate-limiting amino acid for the synthesis of the antioxidant GSH, and thus the function of GPX4, becomes a precious commodity in the TME [12]. Tumor cells often upregulate solute carrier family 7 member 11 (SLC7A11) to gain a competitive advantage over other cells for cystine; this not only blocks tumor ferroptosis, but also deprives T cells of cystine [5]. The lipid peroxidation products 4-hydroxynonenal (4-HNE) and malondialdehyde (MDA) accumulate in the TME, and they provide signals that drive DC maturation, macrophage polarization, and T cell activation towards an immunosuppressive phenotype [13]. Immune cell survival depends on GPX4, but GPX4 also protects tumor cells from immunogenic ferroptosis—the cell death that would otherwise prime the immune system for an antitumoral response [14].

At the tumor core, dead cells leak iron and oxidized lipids into their local environment, creating microenvironments primed for ferroptosis [15,16]. Both bulk transcriptomics and metabolomics ignore these differences, which likely contribute to the lack of success of therapies that trigger ferroptosis [17,18].

There are at least four factors at play in determining where it is drawn: (i) the rate of peroxide generation, determined by membrane PUFA content, lipoxygenase activity and free iron; (ii) the level of catalytic activity provided by GPX4 itself, which depends on its level of expression and post-translational modification, and the availability of selenium cofactors; (iii) whether the GPX4 cofactor, GSH, can be maintained, which depends on cystine/cysteine supply and NADPH; and (iv) the presence of repair back-up systems, particularly FSP1-coenzyme Q10 (CoQ10) and GCH1-BH4, that delay cell death when GPX4 gets overwhelmed [19,20]. In cancer cells, GPX4 is often boosted by transcription or palmitoylation. This means the cell has a higher threshold: it is spared from oxidative stress that would destroy a normal cell [21]. CD8^+^ T cells require GPX4 to prevent ferroptosis during their proliferation and killing phases, but their threshold is weak: when the cell’s ability to regenerate GSH is compromised, its margin of safety falls [22]. The case of DCs is different. Oxidized TME lipids inhibit their antigen presentation and maturation, so the threshold for initiating an immune response increases even if the cells themselves live [13]. Hypoxia is a case in point. It deprives GPX4 of cofactors and lowers the threshold, but it also downregulates lipoxygenase, and this would raise the threshold. Activated T cells secrete interferon-γ (IFN-γ), which downregulates SLC7A11. Lactate builds up, lowering the pH of the TME, which confounds the kinetic rate of lipoxygenases and packing of membrane lipids; tumor cells and immune cells may be pushed in opposite directions.

Four interdependent variables define this threshold: peroxide generation rate, GPX4 catalytic capacity, GSH regenerative potential, and parallel rescue pathway activity (Table 1). These variables differ substantially across cell types, creating divergent ferroptosis susceptibilities in the TME.

**Figure 1:**
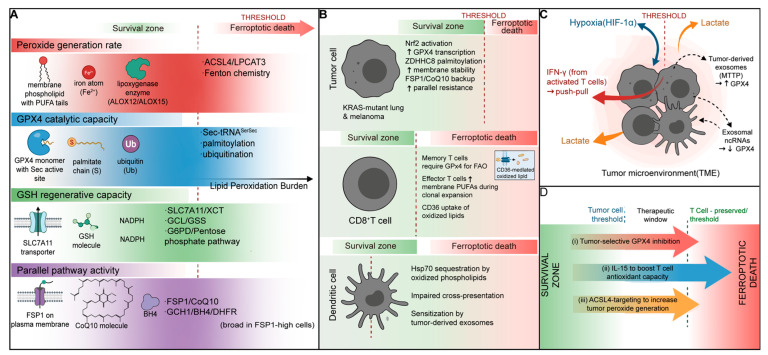
The lipid peroxidation threshold framework. (**A**) Four variables set the threshold in any given cell: the rate of peroxide generation, GPX4 catalytic power, GSH regenerative capacity, and parallel rescue pathways. (**B**) Thresholds differ sharply across TME cell types. (**C**) TME signals retune thresholds dynamically. (**D**) The therapeutic goal—threshold engineering: combination strategies that selectively lower tumor cell thresholds while keeping T cell thresholds intact or even raised, opening a therapeutic window for ferroptosis-based immunotherapy.

We make three main points. First, GPX4 is not a general antioxidant but a specific antioxidant that protects against lethal lipid peroxidation. Second, the results in tumor cell lines are not always predictive of T cell responses. Second, ferroptosis of tumor cells is immunogenic, which releases damage-associated molecular patterns (DAMPs) that activate DCs. Third, effective therapy requires threshold engineering—lowering tumor cell thresholds while preserving or elevating immune cell thresholds. This review integrates enzymology, immunology, and metabolism to provide a comprehensive framework. This integrated model aims to inform refined ferroptosis therapeutic strategies [23]. The translation of ferroptosis to the clinic has been harder than expected. By thinking about the problem in terms of cell-type-specific thresholds, we hope to explain why the drugs have failed, and to outline how a more targeted second-generation ferroptosis drug might look (Figure 1).

A structured literature search was conducted in PubMed, Web of Science, and Scopus from 2012 (formal definition of ferroptosis) to June 2026, using keywords related to GPX4, ferroptosis, TME and immunometabolism. Peer-reviewed original articles and reviews in English were included; preprints and abstracts without datasets were excluded. As a narrative review, no PRISMA flow diagram is provided, and potential selection bias is acknowledged.

## 2. GPX4 as the Lipid Protector of Tumor Cells

**Figure 2:**
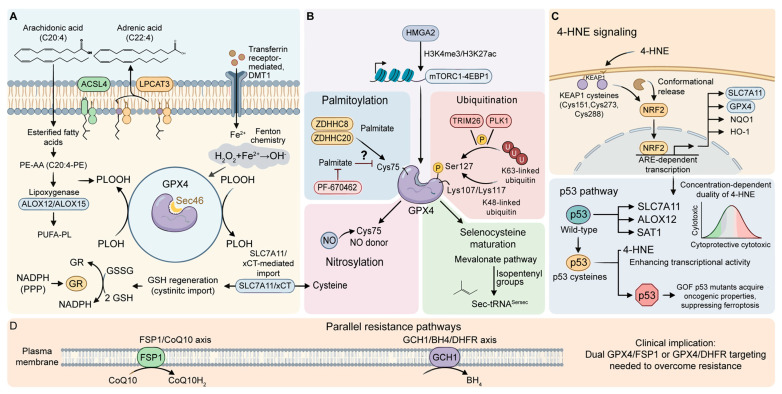
GPX4 molecular mechanisms and regulatory networks. (**A**) The ACSL4/LPCAT3–GPX4 axis: ACSL4 esterifies PUFAs into membrane phospholipids via LPCAT3-mediated sn-2 incorporation22,23. (**B**) Post-translational regulatory network. (**C**) Lipid peroxidation byproduct signaling. (**D**) Parallel resistance pathways.

The textbook view of ferroptosis is a simple pipeline: cystine enters via system Xc−, GSH is synthesized, and GPX4 is the last step, taking out phospholipid hydroperoxides [24]. This is a good model, but also a simplistic one. It makes GPX4 some kind of gatekeeper in an assembly line, when in fact the control is layered, and we are just starting to understand it. In this section, we explore how GPX4 actually regulates tumor cell lipid metabolism. We zero in on the enzymes that prime membranes for peroxidation, the post-translational mechanisms that fine-tune GPX4 activity, and the signaling roles of lipid peroxidation byproducts. These are the areas where the literature still has significant gaps and contradictions [25] (Figure 2).

### 2.1. The ACSL4/LPCAT3-GPX4 Axis

#### 2.1.1. Polyunsaturated Fatty Acids (PUFAs)

ACSL4 incorporates PUFAs, mainly AA (C20:4) and AdA (C22:4), into PE and PC species; LPCAT3 inserts them into the sn-2 position of PE and PC species. LPCAT3 then completes the process to create the lipid pool that ferroptosis consumes [19,26].

#### 2.1.2. Molecular Catalytic Mechanism

Monomeric GPX4 can use phospholipid and cholesterol hydroperoxides as substrates, while tetrameric GPX1-3 can only use H_2_O_2_ [5,27]. Pharmacological inhibition of the Sec active site abrogates all GPX4 catalytic activities, risking off-target effects.

#### 2.1.3. Why This Axis Is Not Enough to Target

Indeed, some tumors express FSP1 so strongly that they resist GPX4 inhibitors [28]. The enrichment of PUFAs does not necessarily sensitize cells to ferroptosis, as SCD1-generated monounsaturated fatty acids can compete for membrane incorporation [22]. Multiple pathways other than the ACSL4/LPCAT3/GPX4 axis may be required for effective ferroptosis therapy [29,30,31].

### 2.2. Post-Translational Modification

Historically, GPX4 regulation has been discussed mostly in terms of gene expression and substrate levels. In addition to transcriptional and substrate-level regulation, GPX4 stability, localization and activity are also regulated by post-translational modifications such as palmitoylation, ubiquitination, phosphorylation, and succination.

#### 2.2.1. Palmitoylation

The reversible addition of palmitic acid to cysteine residues by DHHC-domain containing palmitoyltransferases (ZNFs) is a major regulator of GPX4. Recent studies disagree regarding the responsible enzyme. Zhou et al. (2025) identified ZDHHC8 as the palmitoyltransferase for GPX4 at Cys75, and demonstrated that palmitoylation stabilizes GPX4 and enhances resistance to ferroptosis in melanoma and other cancers [32]. ZDHHC8 expression inversely correlated with CD8^+^ T cell infiltration, and PF-670462 treatment sensitized cells to ferroptosis and enhanced anti-PD-1 efficacy [32]. But Huang et al. (2025) showed that GPX4 is palmitoylated by ZDHHC20, not ZDHHC8, and that ZDHHC20 silencing reduced GPX4 palmitoylation and levels, and heightened ferroptosis [33]. This discrepancy requires resolution. There are several possibilities: different cancer cell lines might use different palmitoylation pathways; GPX4 might be palmitoylated at more than one site, and by different DHHC enzymes (depending on the situation); or there might be technical variations in the detection of palmitoylation. Regardless of the specific enzyme, both studies indicate that palmitoylation stabilizes GPX4 and promotes ferroptosis resistance [32,33].

#### 2.2.2. Ubiquitination and Other PTMs

GPX4 protein levels are also determined by the balance between E3 ubiquitin ligases and deubiquitinases (DUBs). TRIM26 places K63-linked ubiquitin on GPX4 at Lys107 and Lys117, and this actually protects against K48-linked proteasomal degradation [34]. Phosphorylation of TRIM26 at Ser127 by PLK1 further stabilizes the interaction with GPX4, linking the cell cycle to ferroptosis [24]. Conversely, USP8 removes K48-linked ubiquitin from GPX4, blocking proteasomal degradation and maintaining the protein’s stability [24]. In mice, USP8 inhibitors destabilize GPX4, increase sensitivity of cancer cells to ferroptosis inducers, and increase CD8^+^ T cell infiltration and anti-PD-1 efficacy [35]. Unlike direct Sec-targeting inhibitors, upstream modifiers (USP8, ZDHHC8) may offer a wider therapeutic window if differentially expressed between cancer and normal tissues [22]. However, this selectivity remains theoretical pending comprehensive expression profiling in normal human tissues.

#### 2.2.3. Unresolved Questions in GPX4 Regulation

Significant uncertainties remain: upstream signals that induce specific GPX4 modifications are not well understood; crosstalk between palmitoylation and ubiquitination is unexplored; tissue and tumor-specific modification patterns are not characterized; and DHHC enzyme redundancy could limit single-target efficacy. These gaps need to be filled by systematic proteomic and genetic studies.

### 2.3. Lipid Peroxidation Products as Mediators

Lipid peroxidation products are usually considered toxic by-products, but they are now known to be signaling molecules [36]. This signaling aspect complicates the ferroptosis story, as the same peroxidation reactions can lead to cell death, but can also activate cytoprotective gene transcription programs [37].

#### 2.3.1. 4-HNE and MDA

The most well-known lipid peroxidation products that are signaling active are 4-HNE and MDA [3,27]. Low to moderate levels cause modification of protein cysteine, histidine and lysine residues through Michael addition or carbonylation [3]. The effect is concentration-dependent: low levels of 4-HNE might activate adaptive stress responses, while excess 4-HNE induces protein aggregation, mitochondrial dysfunction and death.

This duality is important for cancer: cells that have high basal peroxidation levels may regularly be exposed to low levels of 4-HNE, which maintain adaptive stress responses without causing cell death. This could be the reason why some cancer cells are able to survive at high levels of reactive oxygen species (ROS) without ferroptosis, which has adapted to a range of 4-HNE that keeps Nrf2 activation at non-toxic levels.

#### 2.3.2. Nrf2: Dual Roles in Ferroptosis Regulation

Nrf2 is the main downstream target of lipid peroxidation signaling. Basal levels of KEAP1 target Nrf2 for proteasomal degradation, while electrophilic aldehydes modify KEAP1 cysteine sensors, freeing Nrf2 to promote expression of antioxidant genes such as GPX4 and SLC7A11, creating a negative feedback loop [21].

In cancer, the loss-of-function mutations of KEAP1 (found in KRAS-mutant NSCLC) lead to constitutive activation of Nrf2, which results in resistance to ferroptosis. Importantly, KRAS-G12C inhibitors also induce Nrf2 activation by KEAP1 modification, leading to temporary protection of normal tissues and resistance to tumor cells. The therapeutic challenge is to selectively inhibit the tumor Nrf2 while maintaining its cytoprotective activity in normal tissues [38,39].

#### 2.3.3. Modulation of the p53 Pathway

The regulation of p53 by lipid peroxidation is mutation-dependent. The wild-type p53 can also induce ferroptosis by inhibiting SLC7A11 and inducing ALOX12, SAT1 and iron metabolism genes, and the 4-HNE modification further enhances this activity [24]. Gain-of-function p53 mutations, on the other hand, promote metabolic rewiring, which inhibits ferroptosis. It is still unclear whether lipid peroxidation differentially modulates the wild-type and mutant p53 for selective tumor sensitization [40,41].

#### 2.3.4. Critical Assessment

These results contradict the classical concept of the harmful effect of lipid peroxidation. On the contrary, it is a biphasic signaling system, with acute massive peroxidation promoting ferroptosis and chronic low-level peroxidation promoting adaptive or oncogenic signaling, depending on context [42]. This is why supplementation with non-selective antioxidants has had no success in cancer prevention studies: it can inhibit ferroptosis of pre-cancerous cells and continuously activate Nrf2, which can lead to tumor progression [43,44]. Lipid peroxidation can be viewed as a two-stage process, with acute, massive peroxidation leading to ferroptosis and chronic, low-level peroxidation leading to the production of signaling molecules that can either inhibit or promote tumorigenesis in a context-dependent manner. This is consistent with the identification of ferroptosis as a new cell death program with its own signaling and execution phases [45]. Interestingly, the novel covalent inhibitor A16 (sulfonyl ynamide warhead) has greater selectivity than previous chloroacetamide inhibitors. Additionally, oncogenic regulator HMGA2 upregulates GPX4 via enhancer modification and translational promotion, creating a feedforward resistance loop in pancreatic cancer; HMGA2 targeting may sensitize these tumors to ferroptosis [46].

## 3. GPX4 Is an Immuno-Metabolic Checkpoint

**Figure 3:**
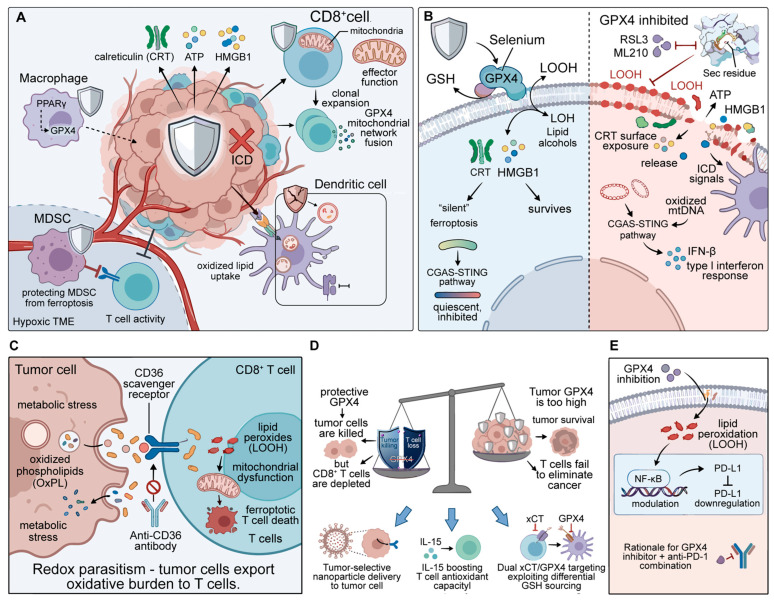
GPX4 as an immuno-metabolic checkpoint across TME cell populations. (**A**) Schematic of GPX4 function across five TME cell types. (**B**) Detail of tumor GPX4–DAMP blockade: intact GPX4 preserves membrane integrity and retains DAMPs intracellularly; GPX4 inhibition triggers lipid peroxide accumulation, membrane rupture, and DAMP/cGAS-STING-mediated innate immune sensing. (**C**) The CD36–GPX4 axis exemplifying “redox parasitism”: tumor-derived oxidized phospholipids are internalized by CD8^+^ T cells via CD36, overwhelming GPX4 antioxidant capacity and triggering T cell ferroptosis. (**D**) The T cell ferroptosis paradox visualized as a balance scale: systemic GPX4 inhibition simultaneously kills tumor cells and depletes protective T cells, with the net outcome determined by which compartment reaches its lipid peroxidation threshold first. (**E**) GPX4–PD-L1 crosstalk: GPX4 inhibition downregulates tumor PD-L1 expression, providing rationale for combination with immune checkpoint blockade, though the mechanistic basis remains unresolved.

It obviously has more to offer than simply blocking cell death via lipid peroxidation. We explore GPX4-mediated immune evasion in tumor cells, CD8^+^ T cells and myeloid populations and test the hypothesis that therapeutic GPX4 inhibition can be selectively targeted to cancer cells while maintaining immune effector function. This multi-cellular landscape is illustrated in Figure 3, and the expression and effect of GPX4 in TME cell types are summarized in Table 2 [47].

### 3.1. GPX4 in Tumor Cells

#### 3.1.1. Inhibition of DAMP Release and Immunogenic Cell Death (ICD)

GPX4 inhibits ICD by inhibiting membrane lipid peroxidation and the subsequent release of DAMPs (CRT, ATP, HMGB1) [48,49]. In TNBC models, GPX4 inhibition leads to ferroptosis that results in higher DAMP release compared to apoptosis, initially indicating high immunogenicity [50]. However, DAMP abundance does not equate to functional immunogenicity, and accumulating evidence challenges the assumption that ferroptotic debris is universally immunostimulatory [51].

#### 3.1.2. Interplay with cGAS-STING

GPX4 also modulates innate immunity via the cGAS-STING pathway. GPX4 inhibition causes mitochondrial membrane damage and mtDNA release, activating cGAS-STING and type I interferon responses that may boost DC cross-priming and NK cell activity [52]. However, this effect is highly context-dependent and remains incompletely characterized. However, this effect is context-dependent: tumors with silenced STING show no interferon response, while chronic low-level STING activation can drive T cell exclusion and ICB resistance. Functional cGAS-STING signaling is a critical determinant of therapeutic outcome [53,54].

#### 3.1.3. GPX4-PD-L1 Connection

*GPX4* knockdown has been reported to decrease tumor PD-L1 expression via lipid peroxidation and NF-κB modulation. In hepatocellular carcinoma (HCC), *GPX4* silencing decreased membrane PD-L1 expression and improved responses to anti-PD-1 antibodies [55]. There has been a prompting interest in combining *GPX4* inhibitors with ICB to simultaneously trigger ferroptosis and alleviate PD-L1-mediated T cell inhibition [56]. But how *GPX4* knockdown leads to PD-L1 reduction remains unclear. Lipid peroxidation can affect protein trafficking and stability, so the decrease in PD-L1 might be a membrane effect rather than true suppression [57]. Moreover, if *GPX4* inhibition concurrently impairs T cell function (Section 3.2), the net effect on antitumor immunity remains uncertain. The GPX4-PD-L1 axis illustrates why targeting a ubiquitous enzyme is problematic: the pleiotropic effects can have a canceling effect [58].

#### 3.1.4. Is Ferroptosis Immunogenic?

An apparent contradiction has emerged in the literature. On the one hand, Efimova et al. and others found that ferroptotic cancer cells protect mice from rechallenge, implying that they are immunogenic [59]. On the other hand, Wiernicki et al. demonstrated that ferroptotic cells inhibit DC activation and fail to induce effective T cell responses [60]. Ferroptotic tumor lysates impaired DC antigen cross-presentation and co-stimulatory capacity compared with apoptotic or necroptotic lysates. This was due to the presence of oxidized phospholipids that directly blocked endosomal processing and loading of peptides onto MHC class I molecules [61]. First, immunogenicity might be dose-dependent: sub-toxic *GPX4* inhibition leading to moderate lipid peroxidation levels may induce ICD pathways without causing the oxidative stress that disorients DCs [62]. Second, cancer cell type is important—cancer lines genetically engineered for *GPX4* overexpression may secrete different types of lipid oxidation products when ferroptotic compared to endogenous tumors. Third, time matters: early ferroptotic cells may still express immunogenic DAMPs on surviving cell membrane blebs, while late post-ferroptotic debris contains immunosuppressive oxidized lipids [62]. The immunological consequences of ferroptosis are not intrinsic to the process, but depend on the levels of oxidants generated, the type of phagocytes that come in contact with the dead cells, and the continued functioning of the antigen presentation machinery. We need thorough studies of the immunological consequences of various methods of inducing ferroptosis and of different cancers. *GPX4* inhibition should not be assumed to reliably promote antitumor immunity via ICD until further validated [63].

### 3.2. GPX4 in CD8^+^ T Cells

#### 3.2.1. GPX4 Is Required for T Cell Survival and Memory

CD8^+^ T cells are the key players in the adaptive antitumor response, and their persistence in the harsh TME depends on antioxidant protection. Mandler et al. were the first to demonstrate that loss of GPX4 in T cells results in spontaneous T cell death exhibiting some ferroptotic features, demonstrating the non-redundant role of GPX4 in T cell survival [64]. Memory T cells are particularly vulnerable to this: they use fatty acid oxidation and thus produce a significant amount of mitochondrial ROS that needs to be kept in check to prevent lipid peroxidation-induced cell death [65]. CD8^+^ T cell survival and function depend critically on GPX4. Activation upregulates membrane PUFA biosynthesis to support clonal expansion, increasing peroxidation vulnerability [66]; memory T cells are particularly dependent on GPX4 due to high mitochondrial ROS from fatty acid oxidation. In the nutrient-deprived, hypoxic TME, sustained antigen stimulation further strains antioxidant defenses. Single-cell transcriptomics shows reduced GPX4 in exhausted CD8^+^ tumor-infiltrating lymphocytes (TILs), though causal direction remains unclear [65].

#### 3.2.2. Oxidized Lipid Scavenging via CD36

Oxidized phospholipids are released by metabolically stressed or ferroptosis-tumor cells. Ma et al. demonstrated that CD8^+^ T cells in the TME express the scavenger receptor CD36, which enables them to take up these oxidized lipids from tumors [67]. The external source of oxidants overcomes T cell GPX4 and triggers ferroptosis, which leads to the death of antitumor effector cells. The CD36-GPX4 interaction establishes a zero-sum game for redox balance where tumor cells, with their generally higher GPX4 expression and cysteine supply via the xCT transporter, compete with T cells [68]. This mechanism is confirmed by the finding that anti-CD36 antibodies can rejuvenate T cells in mice, and suggests that blocking transfer of oxidized lipids may be a more realistic therapeutic strategy than blocking GPX4 itself [67].

#### 3.2.3. The T Cell Ferroptosis Paradox: Therapeutic Implications

A systemic *GPX4* inhibitor will impact both cell types, and the net effect will depend on which cell type reaches its threshold of lipid peroxidation first [65]. Targeted delivery to tumors delivers *GPX4* inhibitors to the tumor while avoiding T cells [45]. Alternatively, co-administration of *GPX4* inhibitors with therapies that increase T cell antioxidant capacity, such as IL-15, may increase the therapeutic index [69]. These are mostly speculative, and the therapeutic window between the toxicity of ferroptosis and T cells is likely to be narrow [70].

### 3.3. GPX4 in Myeloid Cells

#### 3.3.1. DCs

DCs play a key role in antitumor immunity, and cross-presentation is a critical factor in the initiation of adaptive immune responses. This discovery that ferroptotic tumor cells inactivate DCs has far-reaching consequences for GPX4-targeted cancer treatment: while GPX4 loss may induce tumor cell death, the debris may inadvertently paralyze the DCs that are crucial to initiate T cell responses [71]. This is due to oxidized lipids disrupting antigen processing via Hsp70. Hsp70 is a chaperone that shuttles antigens from endosomes to the cytosol for proteasomal degradation and loading onto MHC class I. High-affinity binding of oxidized phospholipids in ferroptotic debris to Hsp70 results in its sequestration in the lipid aggregates and loss-of-function in the cross-presentation pathway [72]. This is a distinct, non-redundant immunosuppressive mechanism that occurs downstream of DAMP recognition—even if DAMPs are released and detected, the antigen processing pathway is obstructed [73]. While DAMP release (Section 3.1.1) and STING activation (Section 3.1.2) have received considerable attention, antigen processing blockade may be equally or more critical for immune outcomes. If ferroptotic debris prevents the DC from presenting tumor antigens, de novo priming of T cells is blocked regardless of the DAMPs released.

#### 3.3.2. Macrophage Polarization

Tumor-associated macrophages (TAMs) are generally skewed to an M2-like phenotype, which facilitates tumor growth, immunosuppression and resistance to therapy. The role of GPX4 in macrophage polarization is intriguing, given that the peroxisome proliferator-activated receptor gamma (PPARγ) ligand, which is a master regulator of M2 polarization, also transcriptionally upregulates antioxidant genes such as those involved in GSH synthesis [74]. It is conceivable that a PPARγ-GPX4 feedback loop contributes to M2 polarization and would make GPX4 a critical regulator of myeloid immunosuppression [75]. The relationship between PPARγ and GPX4 transcription in macrophages is not well documented. The limited reports of GPX4 elevation in M2 macrophages have not determined PPARγ-dependent transcriptional regulation, raising the possibility that GPX4 changes are due to downstream alterations in GSH levels or post-transcriptional regulation. In the absence of such studies, we should be cautious about conclusions that there is a PPARγ-GPX4 axis in TAM biology [76].

#### 3.3.3. MDSCs and Neutrophils

The role of GPX4 in the biology of MDSCs and tumor-associated neutrophils is perhaps the least studied TME populations. They are highly susceptible to oxidative stress and have robust antioxidant systems to maintain their immunosuppressive activity [77]. It has recently been postulated that MDSCs express elevated levels of GPX4 relative to normal myeloid cells, and that this may allow them to survive the harsh oxidatively stressful TME and remain suppressive [78]. If this is true, *GPX4* inhibition would have a two-fold effect of both killing suppressor cells and removing the impediment to T cell activation. But the field has lagged behind in this regard, and the lack of data is emblematic of a general focus on GPX4 biology in tumor cells and T cells, rather than myeloid cells [79]. Insight into the effects of *GPX4* inhibition on DC antigen presentation, macrophage polarization and MDSC apoptosis is needed to assess the net immunogenic effect of therapy.

**Table 2:** GPX4 expression and functional consequences across TME cell types.

GPX4 Expression Level (Relative)	Functional Role and Inhibition Outcome	Key Regulatory Mechanisms	References
High	Role: Prevents ferroptotic death; blocks DAMP release and ICD; maintains membrane integrity for immune evasion. Inhibition outcome: Ferroptotic cell death; CRT exposure; HMGB1 and ATP release; cGAS-STING activation; potential PD-L1 downregulation.	Nrf2-driven transcription; ZDHHC8/ZDHHC20 palmitoylation; HMGA2 activation; SLC7A11-dependent GSH supply	[9,32,33,39,46,48,57,70]
Intermediate to high	Role: Protects against ferroptosis during clonal expansion and effector function; preserves mitochondrial network integrity and oxidative phosphorylation; essential for memory T cell persistence. Inhibition outcome: Spontaneous T cell death with ferroptosis features; accelerated exhaustion; loss of memory formation; impaired fatty acid oxidation.	CD36-mediated oxidized lipid uptake; IL-15 upregulation; NADPH/GSH metabolic constraints; mitochondrial ROS during activation	[22,49,65,67,68,80,81]
Low to intermediate	Role: Preserves membrane integrity for antigen processing; protects Hsp70 from oxidized lipid sequestration. Inhibition outcome: Impaired antigen cross-presentation due to Hsp70 sequestration by oxidized phospholipids; reduced co-stimulatory molecule expression; defective T cell priming.	Exogenous oxidized lipid exposure; limited intrinsic antioxidant reserve; TME hypoxia and acidosis sensitivity	[52,56,61,73]
Moderate (M2 > M1)	Role: Putative PPARγ-GPX4 axis supporting M2-like polarization; general ferroptosis protection. Inhibition outcome: Potential M1 skewing (hypothetical); increased susceptibility to ferroptosis in iron-rich TME niches.	PPARγ transcriptional control (uncertain); iron loading; inflammatory cytokine modulation	[45,48,76]
High	Role: Maintains suppressor cell viability in hypoxic, oxidatively stressed TME; enables sustained immunosuppressive activity. Inhibition outcome: Reduced MDSC survival; relieved T cell inhibition; potential conversion to a less suppressive phenotype.	High basal antioxidant gene expression; hypoxia-adaptive metabolism; Nrf2 pathway engagement	[44,53,74,78,82]
Moderate	Role: Protects against ferroptosis during target cell killing (ROS generation during cytotoxicity); maintains granzyme/perforin-mediated killing capacity. Inhibition outcome: Impaired cytotoxic function; reduced tumor cell killing; potential ferroptotic death under high ROS conditions.	Cytokine activation (IL-2, IL-15); TME metabolite exposure; cystine competition with tumor cells	[22,64,80,81,82,83,84]

### 3.4. Cholesterol 25-Hydroxylase-Oxysterol-EBI2 Axis

Beyond ferroptosis, GPX4 intersects with immune cell trafficking via the Ch25h-oxysterol-EBI2 pathway. Oxysterols act as chemoattractants via EBI2 to guide immune cell positioning, and ferroptosis-derived oxidized lipids may modulate this axis by acting as EBI2 ligands or inhibitors [85,86]. Tumor oxysterols can also induce the recruitment of immunosuppressive Breg and plasmacytoid DCs. From a therapeutic point of view, the inhibition of GPX4 could have a positive or negative effect on the immune system, depending on the oxysterol gradient [87]. The co-administration of GPX4 inhibitors with Ch25h or EBI2 antagonists may be the most effective way to immune position, but needs to be tested experimentally [80]. The mapping of these dynamics will be essential with the help of spatial metabolomics [88].

## 4. Feedback Loops and Spatial Context

**Figure 4:**
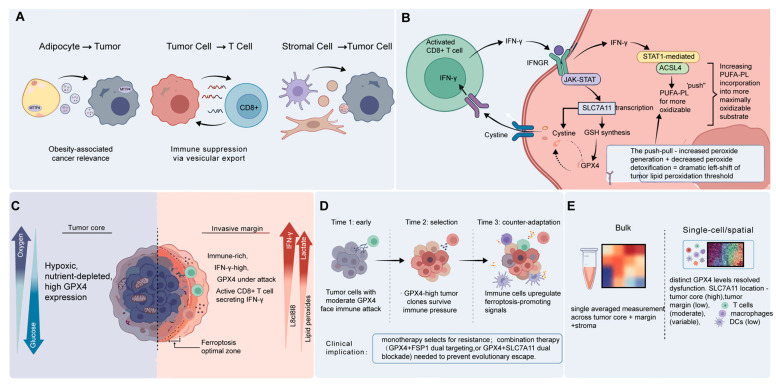
Intercellular feedback circuits and spatial dynamics of GPX4 regulation in the TME. (**A**) Exosome-mediated redox education. (**B**) The IFN-γ push-pull model. (**C**) Spatial zonation: the tumor core selects for GPX4-high, ferroptosis-resistant clones; the invasive margin represents a zone of GPX4 constraint and heightened ferroptosis vulnerability. (**D**) Realistic clinical translation timeline. (**E**) Bulk analysis produces an uninformative population average that obscures spatial heterogeneity, whereas single-cell and spatially resolved methods reveal distinct GPX4 landscapes across cell types and tumor regions.

This is a convenient separation for the purposes of developing a basic understanding, but it conceals a much more complex reality: that is, TME is a highly interlinked signaling network, in which the redox status of one cell population is constantly being modified by its neighbors [82]. Oxidative stress is not only a feature of tumor cells but also a way in which they can modulate the redox state of other cells by releasing vesicles and metabolites, and immune cells can modulate GPX4 activity in tumor cells by releasing cytokines and nutrients. These bidirectional interactions give rise to emergent properties—including spatial patterning, population-level trade-offs in fitness, and evolutionary dynamics—that are not predicted by focusing on either the tumor or immune cell populations in isolation [89] (Figure 4).

### 4.1. Intercellular Feedback Loops

#### 4.1.1. Exosome-Mediated GPX4 Regulation

Extracellular vesicles mediate non-cell-autonomous GPX4 regulation. Adipocyte-derived exosomes carrying MTTP upregulate tumor GPX4 and confer ferroptosis resistance, a key mechanism in obesity-associated cancers [90]. Conversely, tumor-secreted exosomal non-coding RNAs post-transcriptionally suppress T cell GPX4, increasing their ferroptosis susceptibility. Thus, regional redox state reflects the sum of intercellular vesicular signals, not just cell-intrinsic properties.

#### 4.1.2. Immune Cell IFN-γ

Activated CD8^+^ T cells secrete IFN-γ, which downregulates tumor SLC7A11 to restrict cystine uptake and impair GPX4 function, forming an endogenous antitumor ferroptosis pathway [81]. However, cystine is also critical for T cell GSH synthesis and proliferation, creating metabolic competition in the TME [81]. Systemic IFN-γ is limited by toxicity, and sustained exposure induces resistance via alternative antioxidant programs.

#### 4.1.3. The “Push-Pull” Model

In addition to downregulating SLC7A11, IFN-γ induces ACSL4 expression, thereby increasing the substrate pool for lipid peroxidation while simultaneously impairing peroxide detoxification. Liao et al. demonstrated that IFN-γ concurrently induces ACSL4 expression in cancer cells [91]. Inhibition of GPX4 may not be sufficient to induce ferroptosis in tumor cells that have low ACSL4 expression, or low PUFA supply, as is often the case for many tumor types, or in nutrient-poor zones of the TME [92]. Alternatively, increased ACSL4 expression in the absence of *GPX4* inhibition may merely increase the cellular pool of phospholipids, without escalating lipid peroxidation. It is the activation of both pathways, to increase the capacity for lipid peroxidation, and the inability to detoxify peroxides, that drives the ferroptotic threshold down to the point of cell death. This model also explains why IFN-γ-secreting infiltrates drive ferroptosis in some but not all cancers. This effect is contingent on simultaneous activation of both pathways. Patient selection based on high baseline ACSL4 expression, combined with stimuli promoting lethal lipid peroxidation, may be preferable to indiscriminate *GPX4* inhibition. These tumors would then be sensitized for ferroptosis, and may require only modest *GPX4* inhibition to trigger cell death. In this scenario, biomarker-based stratification of patients based on the ACSL4/GPX4 expression ratio could enhance the therapeutic window of ferroptosis-based treatments.

### 4.2. Heterogeneity

#### 4.2.1. GPX4 Zonation

Spatial transcriptomics and scRNA-seq have revealed striking regional zonation of ferroptosis-related gene expression [93]. GPX4 expression and activity are almost universally high in the hypoxic and malnourished core of solid tumors [94]. In contrast, the invasive margin exhibits divergent GPX4 expression patterns [95] where IFN-γ-producing T cells and inflammatory cytokines downregulate SLC7A11, reducing GSH availability and consequently GPX4 function; whereas the invasive margin may represent a therapeutic vulnerability. These spatial patterns also directly dictate differential drug responses: a therapeutic that effectively kills cells at the invasive margin may have no effect on the core tumor population, allowing tumors to quickly recur from the ferroptosis-resistant residual disease.

#### 4.2.2. The Evolutionary Battle at the Tumor–Immune Margin

Spatial GPX4 patterns are shaped by evolutionary selection at the tumor–immune interface [96]. The evolutionary arms race is driven by immunological pressure acting on clones that maintain GPX4 function through activation of Nrf2, upregulation of SLC7A11, or other pathways [97,98]. This is variable from patient to patient and region to region, which accounts for the differences in response. Monotherapy is likely to become resistant quickly, requiring combination strategies to attack several defense nodes.

#### 4.2.3. Key Insight

This spatial heterogeneity compromises the use of bulk GPX4 measurements as predictive biomarkers because the average of the population obscures the existence of distinct cell states within the regions of tumors [99]. Single-cell and spatial profiling are crucial to identify coexisting GPX4-high and -low subpopulations, and to correlate redox states with immune infiltration [100]. These observations led to the conclusion that there is a “ferroptosis-optimal zone,” which is a narrow dose range where the inhibition of GPX4 causes tumor ferroptosis without affecting antitumor T cells [101]. Tumors remain unaffected below this range, and above it, both types of cells die, thereby eliminating sustained immunity. A key pharmacological challenge is to keep the dose in this range.

### 4.3. Reconciling Apparent Contradictions

Two key controversies are resolved by context dependency. First, the ZDHHC8/ZDHHC20 discrepancy is probably cell-type-specific, multi-site modification, or technical variation; these are not mutually exclusive possibilities. Second, the divergent results on the immunogenicity of ferroptosis can be attributed to dose dependency: moderate peroxidation favors ICD, while an overproduction of oxidized lipids negatively affects the function of DCs. These relationships need to be clarified by systematic titration studies using parallel immune readouts.

## 5. Translation

This chapter offers an evaluation of the reasons drug-based therapy of GPX4 remains at the lab bench. It also examines the technologies that could assist the cause and the potential for combined approaches to finally make GPX4 targets a reality.

### 5.1. Why the Current GPX4 Inhibitors Are Unlikely to Succeed

#### 5.1.1. The Pharmacology of RSL3 and Ferroptosis Inducing 56 (FIN56)

The first generation of *GPX4* inhibitors (RSL3, ML162, ML210 and FIN56) are useful research tools but are not appropriate for clinical development. Most do so through covalent modification of Sec46 (FIN56 degrades GPX4), but the chloroacetamide warheads are broadly off-target [102,103]. One of the main reasons for decades of unsuccessful drug discovery is the flat, shallow active site, which does not have deep hydrophobic pockets for selective non-covalent binding [104,105]. Prodrug strategies enhance cell potency but do not address selectivity or lack of metabolic stability.

#### 5.1.2. Selectivity Paradox

GPX4 is a ubiquitously expressed housekeeping enzyme, with high levels in the kidney, testis, brain and hematopoietic cells, creating a severe therapeutic index problem [22]. Conditional deletion in mice shows renal tubular loss causes spontaneous acute kidney failure, neuronal ablation drives lethal neurodegeneration, and testicular deficiency impairs spermatogenesis [22]. Additionally, systemic inhibition depletes antitumor CD8^+^ T cells, undermining endogenous immune control [106]. This “selectivity paradox” means on-target, off-tumor toxicity exceeds that of most oncology targets [107].

#### 5.1.3. Resistance

Even with selectivity solved, acquired resistance to monotherapy develops readily. The most common mechanism is Nrf2 pathway activation (frequent in NSCLC and HNSCC), which upregulates SLC7A11 and antioxidant genes. Additionally, upregulation of FSP1-CoQ10 and DHODH provides alternative detoxification [70,108]. As with other targeted therapies, monotherapy will likely select for resistant clones within months, necessitating combinations.

### 5.2. Emerging Technologies

In response to the challenges of small-molecule inhibition, the field has shifted its focus to other types of drugs. They all have theoretical benefits, but a sobering view reveals that none have overcome the selectivity issue.

#### 5.2.1. Proteolysis-Targeting Chimera (PROTAC) Degraders

PROTAC degraders catalytically deplete GPX4 via the proteasome, potentially achieving efficacy at lower doses than stoichiometric inhibitors [109]. However, effectiveness depends on cellular VHL abundance, making it ineffective in VHL-deficient tumors—paradoxically, those often most sensitive to ferroptosis [110]. Large molecular weight also limits cell permeability and oral bioavailability, and tissue selectivity remains as challenging as with direct inhibitors [110].

#### 5.2.2. Nanoparticle Delivery

Nanoparticle delivery aims to improve tumor selectivity via physical targeting, with formulations encapsulating *GPX4* inhibitors alone or combined with iron supplements/immune adjuvants [4,22,111]. However, the EPR effect is highly variable, with only a small fraction of the dose reaching tumor tissue; most accumulates in the liver, spleen and RES. Even minor cargo leakage could cause severe systemic toxicity given GPX4’s ubiquitous essential role [111,112,113].

#### 5.2.3. Allosteric Inhibitors

A new approach is to inhibit GPX4 at allosteric sites rather than the active site. The basic premise: if the active site is fairly flat and difficult to target selectively, perhaps there are pockets at remote sites that could regulate GPX4 function or stability via a conformational mechanism. Recent studies have mapped potential allosteric sites on the surface of GPX4, and fragment-based screening efforts are ongoing in a number of academic and industrial laboratories [114].

This strategy is highly speculative. There is no reported, validated allosteric *GPX4* inhibitor with suitable pharmacological properties for in vivo use. The difficulties of allosteric drug discovery, including the low affinity of initial hits, the difficulty of establishing the mechanism and unclear structure-activity relationships, are pervasive in the industry. In the case of GPX4, where even orthosteric inhibition has defied traditional medicinal chemistry approaches, allosteric drug discovery is a long shot with a high risk of failure [115].

#### 5.2.4. Critical Analysis

**Figure 5:**
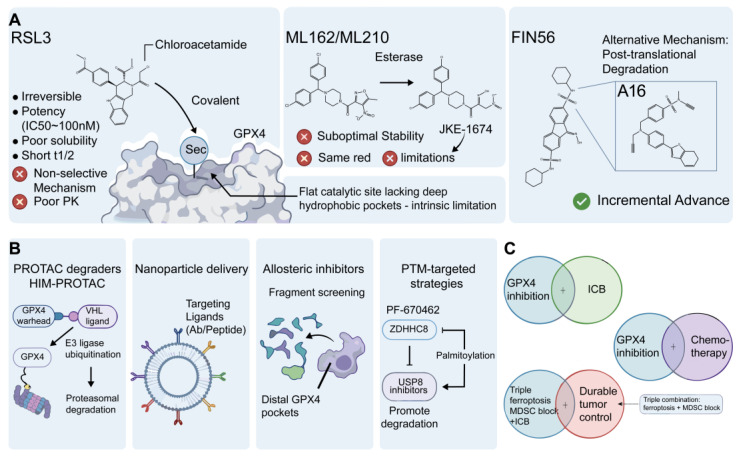
Therapeutic landscape and clinical pipeline for GPX4-targeted therapy. (**A**) Current GPX4 inhibitors and their pharmacological limitations. (**B**) Emerging technology platforms. (**C**) Rational combination strategies.

None of these platforms currently offers a clear clinical path. PROTACs improve potency but not selectivity and depend on E3 ligase expression [105]; nanoparticles address selectivity via delivery but suffer from low tumor accumulation; allosteric inhibitors remain in early discovery [116]. Tumor-targeted nanoparticle co-delivery is the most plausible near-term strategy, but clinical proof-of-concept remains years away [117]. A core takeaway is that target validity does not equal druggability: GPX4 is one of the most challenging targets in contemporary cancer biology (Figure 5).

### 5.3. Rational Combination Strategies

Combination strategies have emerged as the current winning paradigm, due to the lack of efficacy of *GPX4* inhibition on its own. The underlying hypothesis is that *GPX4* inhibition might not be the most effective way to kill cells, but rather a “priming” event that sensitizes cells to other cytotoxic or immunogenic stimuli [69,105]. The link between ferroptosis and immune response to tumors has sparked excitement. Animal studies demonstrate that immunogenic ferroptosis can enhance activation and antigen presentation by DCs, which can, in turn, improve the effectiveness of ICB strategies. Inhibiting *GPX4* in cancer cells leads to increased release of lipid peroxidation products that can act as DAMPs to trigger innate immune response pathways [118,119]. But the relationship between ferroptosis and immunity is more complex than initially thought. As discussed above, GPX4 is essential for T cell survival in the TME, especially for activated CD8^+^ T cells that generate ROS as a consequence of their effector functions [65,120]. Systemic GPX4 suppression might thus affect the immune cells that ICB seeks to stimulate. This dilemma might be circumvented by tumor-selective GPX4 targeting (leaving infiltrating T cells alone) or by dose titrations to enable transient *GPX4* inhibition, which will trigger ICD, while leaving T cells functional. The time course of these competing effects has not been formally mapped and is an important unknown [69].

Tumors resistant to chemotherapy often hyperactivate antioxidant mechanisms. In these situations, *GPX4* inhibition might be used as a “resistance breaker”, increasing responsiveness to chemotherapy [121]. Platinum-resistant ovarian cancers, for instance, have increased GPX4 levels that are associated with ferroptosis resistance, and their combination with platinum compounds has resulted in synergistic killing of cancer cells in preclinical studies [122]. Likewise, temozolomide-resistant glioblastoma cells show upregulation of SLC7A11 and GPX4, and combined targeting of these nodes restores sensitivity to temozolomide-induced cell death. The drug development challenge will be to administer *GPX4* inhibitors safely in combination with highly toxic chemotherapy drugs that already cause considerable normal tissue injury. The additive or potentiating toxicity of combination *GPX4* inhibition plus platinum agents, taxanes or alkylating agents has not been fully investigated in preclinical toxicology studies, and that represents a critical step towards clinical development of combination therapies [123].

In n HCC models, *GPX4* inhibition alone induces recruitment of immunosuppressive MDSCs through lipid peroxidation chemotaxis, which leads to tumor growth. Triple combination (*GPX4* inhibition + MDSC blockade + anti-PD-1) is effective in inducing durable control of disease when dual combination fails. This highlights the importance of patient stratification: myeloid-rich tumors require MDSC-targeting components, while T cell-inflamed tumors benefit most from GPX4 + ICB [124,125,126] (Table 3).

### 5.4. Who Will Benefit from GPX4-Targeted Therapy?

Patient selection will be critical. Sensitive tumors typically have high baseline lipid peroxidation, active PUFA metabolism, low compensatory pathway expression, and wild-type p53. Resistant tumors show activated Nrf2, high FSP1, or abundant MDSCs [120,127]. First-in-human trials of next-generation agents are unlikely before the late 2020s, with clinical success possible only in the 2030s. In the near term, the greatest value comes from integrating ferroptosis biology into existing therapies rather than direct GPX4 targeting [120,127].

## 6. Biomarkers for Patient Population Stratification

The biggest impediment to clinical development of GPX4-targeted therapies is surprisingly simple: we still lack biomarkers to predict who will benefit from therapy. There are myriad prognostic associations between GPX4 and survival in various cancers, but a biomarker that is prognostic is not necessarily predictive of response to a particular targeted therapy. This is a critical point in clinical trial design, which is often conflated in much of the literature on ferroptosis, creating an illusion that we are closer to meaningful patient stratification than the evidence warrants. Here, we provide a critical evaluation of the state of current biomarkers, identify the technical and intellectual hurdles to their development, and suggest a path forward for rational biomarker development [128].

### 6.1. GPX4 Expression

#### 6.1.1. Pan-Cancer Expression Patterns

GPX4 is generally over-expressed in a wide range of cancers, including prostate adenocarcinoma, lung adenocarcinoma, colorectal carcinoma and acute myeloid leukemia (AML), when compared to adjacent normal tissues [58]. This expression pattern has given rise to a plethora of bioinformatics studies correlating increased GPX4 expression with decreased overall survival, and with that, the persistent belief that GPX4 represents a promising therapeutic target for a broad range of cancer types [129,130].

However, these pan-cancer associations require cautious interpretation. GPX4 is a critical enzyme for cell survival; its induction in highly proliferative cancer cells could reflect increased metabolic demands, rather than induction of a specific pathway targeted by ferroptosis. To sort these two scenarios apart requires functional experiments that go well beyond transcriptomic correlation studies, and there are few of these in the literature [129].

#### 6.1.2. AML and Prostate Cancer

AML may represent the best case for GPX4 as a tumor biomarker. Several studies have found that elevated GPX4 expression (both mRNA and protein) is associated with poor patient outcomes and clearly highlights a group of patients whose tumor cells are GPX4-dependent [60]. Most importantly, co-expression of GPX4 and AIFM2 (the gene encoding FSP1) defines a subset with especially poor prognosis, suggesting the possibility that tumors that are dependent on both parallel pathways of ferroptosis suppression may be the most sensitive to disruption of this pathway. The therapeutic implications here are encouraging: AML cells with defective electron transport chains are highly sensitive to *GPX4* inhibition, suggesting a mitochondrial-lipid–redox axis that could be targeted in the clinic [131].

The prostate cancer biomarker space, on the other hand, demonstrates the potential and the perils of expression-based biomarker discovery. A recent report identified a four-protein signature in serum (GPX4, NDUFS4, PRDX5 and TXNRD2) that was predicted with an AUC of 0.988 using extreme gradient boosting for castration-resistant prostate cancer (CRPC) [132]. This is an extraordinary outcome, but it should be taken with a pinch of salt. This model was developed and tested with only 28 serum samples from CRPC patients, which is not enough to properly develop and validate the model. The so-called “independent” cohort of 11 CRPC cases was used in the study. Complex machine learning models tend to produce overinflated AUC values in small groups of patients, and the absence of any “external” validation in a prospective, geographically separate cohort means this signature is not even close to being clinically ready. Further, all four proteins in the panel are part of the broader cellular antioxidant system; their upregulation could be merely due to increased oxidative stress in the disease, rather than specific ferroptosis addiction. Although the association between these biomarkers and time to CRPC is consistent with the pathway, these data cannot progress beyond hypothesis generation until they are confirmed in properly sized prospective cohorts [133].

#### 6.1.3. Prognostic vs. Predictive Biomarkers

A critical limitation is that prognostic biomarkers (correlating with survival) are not necessarily predictive of therapeutic response. High GPX4 expression is consistently prognostic of poor outcome, but does not guarantee sensitivity to inhibition. Validated predictive biomarkers must reflect pathway dependency rather than expression alone, for example, the ACSL4/GPX4 activity ratio, or sensitivity in the context of compensatory pathway inhibition. Expression levels also do not reflect functional pathway activity: a tumor with high GPX4 but concurrent FSP1 upregulation may be fully resistant, while moderate GPX4 with limited substrate availability may also be unresponsive. The field urgently needs markers of ferroptosis dependency rather than simple expression abundance [134].

### 6.2. Lipid Peroxidation Metabolomics

#### 6.2.1. Plasma Lipid Peroxides as Dynamic Biomarkers

Lipid peroxidation metabolites offer dynamic, functional readouts of pathway activity, making them promising pharmacodynamic markers [5]. However, practical challenges limit clinical use: metabolites are highly labile and prone to ex vivo artifactual generation; inter-laboratory measurement variability is high [135]; and lipid peroxidation is not specific to ferroptosis. Pre-analytical standardization remains the largest barrier [136].

#### 6.2.2. MS Imaging for Tumor Lipid Landscapes

Mass spectrometry imaging (MSI) of tumor sections is an alternative approach, visualizing the distribution of lipid peroxidation products with high spatial resolution, providing key information about the tumor microenvironment. This approach has revealed wide variability in lipid peroxidation across tumors, with ferroptosis-susceptible areas often co-localized with immune cells, rather than spreading homogeneously throughout the malignant tissue. Clearly, this knowledge could inform biopsy-based patient selection. However, MSI is currently limited to research labs, requires fresh-frozen tissues, and is slow and thus impractical for clinical use. It is unclear whether these challenges can be addressed through faster workflows and large, clinically annotated biobanks.

### 6.3. A Multi-Analyte Biomarker

#### 6.3.1. Multi-Omic Integration

Single analytes cannot capture ferroptosis complexity, and multi-omic ferroptosis scores integrating pro- and anti-ferroptotic genes show greater predictive value. In melanoma, a high ferroptosis score strongly predicts anti-PD-1 response, reflecting tumor–immune ferroptosis crosstalk [137,138]. Notably, post-translational modifications such as ZDHHC8-mediated palmitoylation may be more predictive than expression levels [137]. Low GPX4 with high palmitoylation can confer greater resistance than high GPX4 with low modification [137].

#### 6.3.2. Single-Cell Biomarker Discovery

Single-cell profiling reveals cell-type-specific ferroptosis heterogeneity, explaining inconsistent bulk biomarker performance. It enables two key applications: identifying tumor clones with active ferroptosis defense, and stratifying patients at risk of T cell depletion during therapy [137]. Overall, the biomarker deficit is the largest barrier to clinical translation [139,140]. A three-stage roadmap is proposed: Stage 1—pharmacodynamic target engagement assays; Stage 2—predictive signature validation for patient selection; Stage 3—resistance marker identification. Rigorous prospective validation is essential to avoid the biomarker failures of other targeted therapy fields [83,84].

## 7. Conclusions

The previous sections have presented a complex picture. GPX4 is not a binary switch; altering it has different effects in cancers, T cells, and myeloid subpopulations, hence the difficulties with conventional drug discovery. Lipid peroxidation levels vary across compartments, pharmacological development has reached an impasse, and we are not yet able to identify the right patients. This last part reveals the questions we have to tackle and proposes a roadmap for the development of precision ferroptosis medicine therapies.

Current GPX4 interactome data derive almost exclusively from cancer cell lines, with no cell-type-specific maps for T cells, DCs or macrophages in the TME. Cell-type-specific interactors could be exploited for selective therapeutic targeting. Proximity labeling assays in isogenic systems under physiologically relevant TME conditions are required to build comprehensive interactome atlases. GPX4 fin-loop mutations identified in cancer alter catalytic activity, substrate specificity and drug sensitivity, challenging the default assumption of universal wild-type expression. Patient-derived variants should be cataloged from cancer genome databases and functionally characterized in isogenic cell lines to refine patient stratification. GPX4 exerts non-canonical functions in transcription regulation and mitochondrial dynamics independent of its peroxidase activity. Catalysis-only inhibitors cannot fully recapitulate genetic knockout phenotypes. Separation-of-function mutants are needed to disentangle canonical and non-canonical effects across cell types.

Three technological advances are critical to bridge basic biology and clinical translation: single-cell, spatially resolved imaging of GPX4 protein abundance, post-translational status and activity in human tumor biopsies. Cell-permeable activity-based probes for real-time in vivo monitoring of GPX4 catalytic state as pharmacodynamic markers. Tumor-selective delivery systems with validated tumor-to-normal tissue drug concentration ratios show greater therapeutic success. Three design principles should guide early-phase clinical trials: Mandatory serial biopsy with multi-omic biomarker profiling embedded in all Phase I trials for prospective qualification. Adaptive trial designs from Phase Ib onward, enabling biomarker-driven patient enrichment based on interim response data. Expansion cohorts dedicated to rational combination regimens tailored to tumor–immune phenotypes. We propose a three-phase roadmap for precision ferroptosis medicine: Phase 1 (Present–2028): Foundation building–generate single-cell GPX4 activity atlases of human tumors; develop cell-permeable activity probes; characterize clinical GPX4 variants; map cell-type-specific interactomes; optimize tumor-targeted delivery systems. Phase 2 (2028–2033): Clinical translation–initiate first-in-human trials of second-generation agents with embedded biomarker validation; test combination regimens, prioritizing triple therapy (ferroptosis induction + MDSC blockade + ICB) for myeloid-infiltrated tumors. Phase 3 (2033 onward): Personalized medicine–implement patient-specific threshold profiling via biopsies and circulating markers, with tailored GPX4 modulation to push tumor cells past their peroxidation threshold while preserving immune function.

## Figures and Tables

**Table 1:** Proposed experimental readouts for each variable are as follows:

Variable	Recommended Readouts
Peroxide generation	C11-BODIPY581/591 fluorescence; LC-MS/MS of oxidized phosphatidylethanolamines (oxPE); expression of acyl-CoA synthetase long-chain family member 4 (ACSL4) and lysophosphatidylcholine acyltransferase 3 (LPCAT3)
GPX4 catalytic capacity	Direct GPX4 activity assay (NADPH-coupled); selenoprotein quantification; acyl-biotin exchange for palmitoylation status
GSH regenerative capacity	GSH/GSSG ratio; cystine uptake flux; NADPH/NADP^+^ ratio
Parallel rescue pathways	FSP1 expression/activity; CoQ10 redox state; dihydroorotate dehydrogenase (DHODH) pharmacological sensitivity

**Table 3:** GPX4-targeted therapeutics.

Compound/Strategy	Mechanism	Clinical Stage and Key Limitation	Selectivity Profile	Main Toxicity Concern	Clinical Barrier
RSL3	Covalent Sec46 inhibition (chloroacetamide)	Research tool. Flat active site; no druggable binding pocket; electrophilic warhead reactivity; poor pharmacokinetics.	Non-selective (broad cysteine reactivity); affects the kidney, testis, CNS, and T cells.	Kidney, testis, CNS, T cell depletion	Flat active-site architecture; off-target binding
ML162	Covalent Sec46 inhibition (chloroacetamide)	Research tool. Same class limitations as RSL3.	Non-selective; affects the kidney, testis, CNS, and T cells.	Kidney, testis, CNS, T cell depletion	Flat active-site architecture
ML210	Prodrug of JKE-1674 (esterase-cleaved chloroacetamide)	Research tool. Covalent mechanism preserved; metabolic instability; prodrug improves cell potency only.	Non-selective; affects the kidney, testis, CNS, and T cells.	Kidney, testis, CNS, T cell depletion	Same selectivity challenges as RSL3
A16	Sulfonyl ynamide-based covalent inhibition	Lead optimization. In vivo PK/PD uncharacterized; long-term ynamide stability uncertain.	Improved over chloroacetamides (pancreatic models); affects the kidney, testis, CNS, and T cells.	Kidney, testis, CNS, T cell depletion	Ynamide stability; in vivo characterization pending
PF-670462 (ZDHHC8 inhibitor)	Blocks ZDHHC8-mediated GPX4 palmitoylation	Target validation. Cell-type-specific palmitoylation landscapes unmapped; ZDHHC8 vs. ZDHHC20 specificity unresolved.	Theoretical selectivity for ZDHHC8-dependent tumors; off-target palmitoylation possible.	Off-target palmitoylation effects	ZDHHC8 vs. ZDHHC20 specificity unresolved
USP8 inhibitors	Promotes K48-linked ubiquitination and GPX4 degradation	Target validation. Tissue-dependent USP8 function; multiple substrates beyond GPX4; requires normal tissue profiling.	Theoretical tumor selectivity is higher USP8-GPX4 dependency in cancer.	Multiple USP8 substrate effects	Comprehensive normal tissue profiling needed
HIM-PROTAC	Bifunctional VHL-recruiting degrader	Proof-of-concept. VHL dependency; large molecular weight; limited oral bioavailability and cellular permeability.	Kidney (VHL-expressing tubules); catalytic degradation improves potency but not tissue selectivity.	Kidney tubular toxicity	VHL dependency; large molecular weight; permeability
Nanoparticle-RSL3	Liposomal or micelle-encapsulated RSL3	Formulation development. EPR variability; majority cleared by RES; manufacturing complexity; targeting ligand optimization ongoing.	Tumor selectivity via physical targeting (low tumor dose fraction); liver and spleen (RES) accumulation.	Liver, spleen (RES) toxicity; leakage-induced systemic toxicity	EPR variability; low tumor dose fraction
GPX4-i + ICB	Tumor ferroptosis + T cell checkpoint release	Preclinical (mouse models). Optimal dosing schedule undefined; T cell toxicity at tumor-effective doses.	Theoretical dose separation; depends on transient vs. sustained inhibition.	T cell depletion; loss of ICB efficacy	Optimal dosing schedule undefined
GPX4-i + Chemotherapy	Resistance breaking in platinum/temozolomide-resistant tumors	Preclinical. Toxicology of combinations uncharacterized; additive normal tissue toxicity.	Tumor-selective if cancer has a higher oxidative load.	Bone marrow, renal, and neurological toxicity	Additive normal tissue toxicity; combination toxicology uncharacterized
Triple (GPX4-i + MDSC block + ICB)	Tumor ferroptosis + suppressor blockade + T cell activation	Preclinical (HCC models). Addresses tumor–immune ecosystem; hepatotoxicity; immune-related adverse events.	Biomarker-defined patient selection is needed.	Hepatotoxicity; immune-related adverse events	Three-drug toxicity management; sequencing undefined

## Data Availability

No new data were created or analyzed in this study. Data sharing is not applicable to this article.

## Abbreviations

The following abbreviations are used in this manuscript:

4-HNE	4-hydroxynonenal
4EBP1	Eukaryotic translation initiation factor 4E-binding protein 1
AA	Arachidonic acid
ACSL4	Acyl-CoA synthetase long-chain family member 4
AdA	Adrenic acid
AIFM2	Apoptosis-inducing factor mitochondria-associated 2
ALOX12	Arachidonate 12-lipoxygenase
ALOX15	Arachidonate 15-lipoxygenase
AML	Acute myeloid leukemia
APEX2	Ascorbate peroxidase 2
ARE	Antioxidant response element
ATP	Adenosine triphosphate
AUC	Area under the curve
A16	Sulfonyl ynamide-based covalent GPX4 inhibitor A16
BH4	Tetrahydrobiopterin
BioID	Biotin identification
Breg	Regulatory B cell
ccRCC	Clear cell renal cell carcinoma
cGAS	Cyclic GMP-AMP synthase
Ch25h	Cholesterol 25-hydroxylase
CoQ10	Coenzyme Q10
CRPC	Castration-resistant prostate cancer
CRT	Calreticulin
CUL3	Cullin 3
CXCR2	C-X-C chemokine receptor type 2
DAMPs	Damage-associated molecular patterns
DCs	Dendritic cells
DHFR	Dihydrofolate reductase
DHODH	Dihydroorotate dehydrogenase
DUBs	Deubiquitinases
EBI2	Epstein–Barr virus–induced G protein-coupled receptor 2
EPR	Enhanced permeability and retention
erastin	Ferroptosis inducer erastin
FIN56	Ferroptosis-inducing 56
FSP1	Ferroptosis suppressor protein 1
GCH1	GTP cyclohydrolase 1
GENIE	Genomics Evidence Neoplasia Information Exchange
GPX4	Glutathione peroxidase 4
GR1	Granulocyte differentiation antigen 1
GSH	Glutathione
GSSG	Glutathione disulfide
HCC	Hepatocellular carcinoma
HIF-1α	Hypoxia-inducible factor 1α
HIFs	Hypoxia-inducible factors
HIM-PROTAC	GPX4-targeting proteolysis-targeting chimera HIM-PROTAC
HMGB1	High mobility group box 1
HMGA2	High mobility group AT-hook 2
HNSCC	Head and neck squamous cell carcinoma
HO-1	Heme oxygenase 1
Hsp70	Heat shock protein 70
ICD	Immunogenic cell death
ICB	Immune checkpoint blockade
IFN-γ	Interferon-γ
IL-15	Interleukin-15
IL-2	Interleukin-2
JAK	Janus kinase
JKE-1674	Active metabolite of ML210
KEAP1	Kelch-like ECH-associated protein 1
kcat/KM	Catalytic efficiency
LC-MS/MS	Liquid chromatography-tandem mass spectrometry
LPCAT3	Lysophosphatidylcholine acyltransferase 3
LPS	Lipopolysaccharide
MDA	Malondialdehyde
MDSCs	Myeloid-derived suppressor cells
MHC	Major histocompatibility complex
ML162	Covalent GPX4 inhibitor ML162
ML210	Covalent GPX4 inhibitor ML210
MSI	Mass spectrometry imaging
MSK-IMPACT	Memorial Sloan Kettering-Integrated Mutation Profiling of Actionable Cancer Targets
MTTP	Microsomal triglyceride transfer protein
mTORC1	Mechanistic target of rapamycin complex 1
NADPH	Nicotinamide adenine dinucleotide phosphate
NFE2L2	Nuclear factor, erythroid 2-like 2
NK cells	Natural killer cells
NQO1	NAD(P)H quinone dehydrogenase 1
Nrf2	Nuclear factor erythroid 2-related factor 2
NSCLC	Non-small cell lung cancer
OS	Overall survival
PC	Phosphatidylcholine
PD-1	Programmed cell death protein 1
PD-L1	Programmed death-ligand 1
PE	Phosphatidylethanolamine
PF-670462	ZDHHC8 inhibitor PF-670462
PFS	Progression-free survival
PLK1	Polo-like kinase 1
PPARγ	Peroxisome proliferator-activated receptor gamma
PROTACs	Proteolysis-targeting chimeras
PUFAs	Polyunsaturated fatty acids
RES	Reticuloendothelial system
ROS	Reactive oxygen species
RSL3	Ras-selective lethal 3
SAR	Structure-activity relationship
SAT1	Spermidine/spermine N1-acetyltransferase 1
Sec	Selenocysteine
shRNA	Short hairpin RNA
SLC7A11	Solute carrier family 7 member 11
S6K	Ribosomal protein S6 kinase
STAT1	Signal transducer and activator of transcription 1
STING	Stimulator of interferon genes
system x_c^−^	Cystine/glutamate antiporter system x_c^−^
TAMs	Tumor-associated macrophages
TCGA	The Cancer Genome Atlas
TILs	Tumor-infiltrating lymphocytes
TME	Tumor microenvironment
TNBC	Triple-negative breast cancer
TRIM26	Tripartite motif containing 26
Treg cells	Regulatory T cells
TRM	Tissue-resident memory T cells
Ub	Ubiquitin
USP8	Ubiquitin-specific peptidase 8
VHL	Von Hippel-Lindau
ZDHHC8	Zinc finger DHHC-type palmitoyltransferase 8
ZDHHC20	Zinc finger DHHC-type palmitoyltransferase 20
